# Defecation enhances cerebral perfusion and delays fatigue in elite triathletes

**DOI:** 10.1080/15502783.2023.2206380

**Published:** 2023-04-27

**Authors:** Chen-Chan Wei, Giancarlo Condello, Ai-Lun Yang, Szu-Hsien Yu, Yi-Hung Liao, Chung-Yu Chen, Chi-Chieh Hsu, Chi-Yang Huang, Chia-Hua Kuo

**Affiliations:** a University of Taipei, Laboratory of Exercise Biochemistry, College of Kinesiology, Taipei, Taiwan; b University of Parma, Department of Medicine and Surgery, Parma, Italy; c National Ilan University, Department of Leisure Industry and Health Promotion, I-Lan, Taiwan; d National Taipei University of Nursing and Health Sciences, Department of Exercise and Health Science, Taipei, Taiwan; e Buddhist Tzu Chi Medical Foundation, Cardiovascular and Mitochondrial Related Disease Research Center, Hualien Tzu Chi Hospital, Hualien, Taiwan; f Department of Medical Research, China Medical University Hospital, China Medical University, Taichung, Taiwan; g China Medical University, Graduate Institute of Biomedical Sciences, Taichung, Taiwan; h Physical Education and Sports School, Soochow University, Suzhou, China

**Keywords:** Constipation, stool, abdominal distension, *dantian*, gut-brain axis

## Abstract

**Background:**

Rectal distension increases regulatory burden to autonomic nervous system in the brain.

**Purpose:**

To determine the effect of rectal defecation on endurance performance and blood supply to the prefrontal brain and sub-navel regions of elite triathletes.

**Methods:**

Thirteen elite triathletes completed a cycling time trial (80% VO_2max_) under defecated and non-defecated conditions, using a counterbalanced crossover design. Oxygenation and blood distribution in prefrontal brain and sub-navel regions were monitored by near-infrared spectroscopy (NIRS) during cycling.

**Results:**

Defecation moderately decreased systolic blood pressure (−4 mmHg, *p* < 0.05, d = 0.71), suggesting an alleviation of autonomic nervous activity. During the exercise trials, fatigue (cycling time to exhaustion) occurred when cerebral oxygenation decreased to ~ 5 % below baseline regardless of treatment conditions, suggesting a critical deoxygenation point for sustaining voluntary exertions. Cerebral blood (estimated by total hemoglobin) increased progressively throughout the entire exercise period. Defecation decreased sub-navel oxygenation levels below the non-defecated level, suggesting an increased sub-navel oxygen consumption. Exercise also decreased sub-navel blood distribution, with minimal difference between non-defecated and defecated conditions. Defecation improved blood pooling in the prefrontal brain during exercise (*p* < 0.05) and enhanced cycling performance in triathletes (Non-defecated: 1624 ± 138 s vs. defecated: 1902 ± 163 s, d = 0.51, *p* < 0.05).

**Conclusion:**

Our results suggest that improved exercise performance after defecation is associated with greater blood availability to compensate deoxygenation in the prefrontal brain region during exercise. Further investigation is needed to examine the role of increasing sub-navel oxygen consumption in the performance improvement after defecation.

## 1. Introduction

Muscle contractions are primarily controlled by central commands from the brain that recruit motor neurons [1,2]. As the prefrontal brain becomes more active during exercise, there is an increasing metabolic need that must be met by an increase in cerebral blood supply to maintain oxygen levels in the brain [3]. Maintaining adequate amount of oxygen in the brain is an important factor that limits a person’s capability to sustain high-intensity exercise [4]. Additionally, the enteric nervous network, which extends from the gastrointestinal tract to the anal canal [5], can be affected by rectal distension, potentially increasing regulatory burden to distract blood supply to the prefrontal brain [6]. Thus far, it remains to be examined whether rectal defecation can spare more blood for the prefrontal brain to recruit muscles during high-intensity exercise.

To address the questions regarding how exercise impacts blood distribution to the central nervous system versus enteric nervous system, and whether rectal defecation can affect the hemodynamic response induced by exercise, we measured real-time changes in blood distribution and oxygenation using NIRS in both the prefrontal brain and sub-navel regions during cycling to exhaustion trials under defecated and non-defecated conditions. Since glucose is the primary fuel for the nervous system, we used an 18F-fluorodeoxyglucose positron emission tomography (PET) image to localize the regions with high glucose uptake for NIRS measurement.

## 2. Methods

### 2.1. Ethical approval

The study protocol was approved by University of Taipei Institutional Review Board (trial number: IRB-2019-087). Testing procedures and potential risks involved in the study were informed to all participants before receiving written consent forms.

### 2.2. Participants

Fifteen elite young triathletes (*M* = 9; F = 6) voluntarily participated in the study with two dropouts associated with difficulty to evacuate stool before the cycling trials. The remaining thirteen young triathletes (East Asian, age = 20.3 ± 0.5 y, height = 167.0 ± 2.4 cm, body mass = 64.3 ± 2.7 kg) completed the trial (Table 1). These triathletes maintained 20–23 h of swimming, cycling, and running each week. Self-reported habitual bowel evacuation times of each triathlete were P1, 0900 am; P2, 0850 am; P3, 0900 am; P4, 0900 am; P5, 0600 am; P6, 0535 am; P7, 0900 am; P8, 0900 pm; P9, 1130 am; P10, 1000 pm; P11, 0900 am; P12, 0500 am; and P13, 0900 am.

**Table 1.** Participant characteristics.

Participant	Sex	Rank	Category	Competing Experience
1	F	Top 3	Elite U23	Asian Championship
2	M	Top 10	Elite U23	World University Triathlon Championship
3	M	Top 3	Elite U23	World University Triathlon Championship
4	F	Top 10	Elite U23	National games Triathlon medalist
5	M	Top 5	Elite U19	World schools Triathlon Championship
6	F	Top 10	Elite U23	National potential triathlon team
7	F	Top 3	Elite U23	World University Triathlon Championship
8	F	Top 3	Elite U23	World University Triathlon Championship
9	M	Top 10	Elite U23	National Potential Triathlon Team
10	M	Top 5	Elite U23	Asian Championship
11	M	Top 20	Elite	Asian Cup
12	M	Top 20	Elite	Asian Cup
13	F	Top 5	Elite U23	World University Triathlon Championship

All participants are ethnic East Asian (age = 20.3 ± 0.5 y) and was competing national teammate at least once. Self-reported habitual defecation times of each triathlete are as follows: Participant 1, 0900 am; Participant 2, 0850 am; Participant 3, 0900 am; Participant 4, 0900 am; Participant 5, 0600 am; Participant 6, 0535 am; Participant 7, 0900 am; Participant 8, 0900 pm; Participant 9, 1130 am; Participant 10, 1000 pm; Participant 11, 0900 am; Participant 12, 0500 am; Participant 13, 0900 am.

### 2.3. Study design

A counter-balanced crossover study of defecated and non-defecated conditions was performed, with a 1-week washout period to ensure the optimal performance with full recovery from the previous test. The experimental procedures started at 0600 am in the laboratory. To compensate water loss during their overnight sleep, a tap water (300 mL) was provided in the morning before bowel evacuation. Half of the participants voluntarily defecated their stool and the rest of participants remained non-defecated. Endurance performance (cycling time to exhaustion at 80% VO_2max_) was conducted 90 min (0730 am) after the defecation at 18.5 Celsius degree and 60% humidity following a 10-h overnight fasting.

### 2.4. Dietary control

All participants were informed not to change dietary habit and avoid nutritional supplements and alcohol 7 days before the study. To minimize potential influence of dietary carbohydrate on performance on day 2 [7], high carbohydrate meals were prepared by a dietitian (total 24-h calorie intake: 35–40 kcal per kg with carbohydrates-to-protein ratio 9: 1) one day before interventions. With inclusion of a 12-h overnight fast, the dietary control period was 36 h for each participant. This dietary control is significantly longer than normal gastrointestinal transit time in humans [8], and is expected to prevent the influence of meal difference on defecation. All participants were informed to avoid additional nutritional supplements, stimulants, and alcohol one week before the study.

### 2.5. BP and arterial stiffness

To assess autonomic nervous activity, blood pressure and resting levels of brachial-ankle pulse wave velocity (baPWV) (Omron Colin VP-1000, Japan) were measured at supine position 10 min after arrival to the laboratory (0610 am) after defecation.

### 2.6. Tissue oxygenation and blood distribution

**Figure 1.**
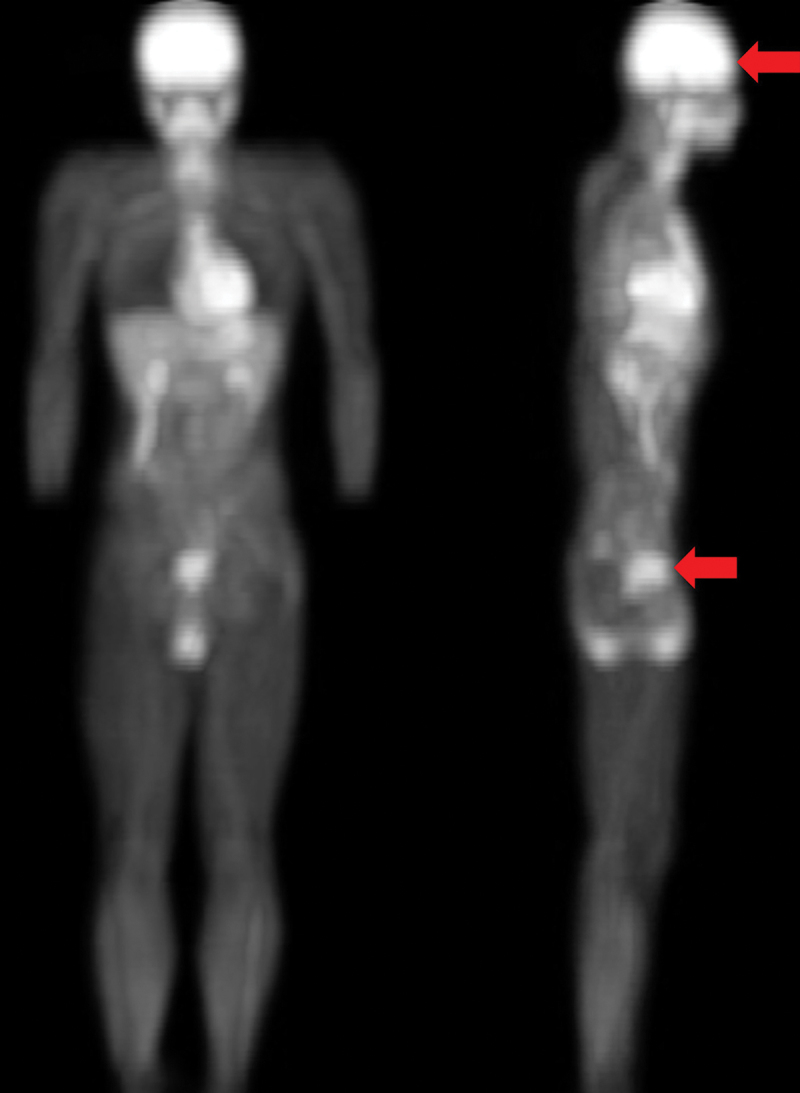
Positioning optical detection probe of near-infrared spectroscopy (NIRS). The whole body 18F-fluorodeoxyglucose positron emission tomography (PET) image indicates the metabolically active sub-navel and cerebral regions where the optical detection probes of near-infrared spectroscopy (NIRS) were placed (indicated by red arrows). PET scan was conducted 1 h after injecting glucose solution containing 18F-fluorodeoxyglucose (400 MBq) by a young participant (24 y) before scanning under overnight fasted condition.

The real-time changes in blood distribution and oxygenation in the sub-navel and prefrontal brain regions were monitored simultaneously by a noninvasive near-infrared spectroscopy (NIRS) using a two-emitters/detector probe [9]. NIRS optical probes were placed on sub-navel region (3 inches below umbilicus) and prefrontal cortex (above the superciliary arch) in the metabolically active regions as indicated by 18F-fluorodeoxyglucose PET image (Figure 1).

Blood distribution in the capillaries of the tissue was optically detected by tracing mobile hemoglobin concentration (oxy- and deoxy-hemoglobin) [10], whilst the oxygenation levels (% oxygen saturation) were calculated as percent oxyhemoglobin to total hemoglobin during the same measuring period [3]. Data were collected at 10 Hz via bluetooth using a wireless NIRS device with Oxysoft software (PortaLite, Artinis Medical System, Elst, Netherlands) during a cycling time to exhaustion test at 80%VO_2max_. This device allows detection encompassing a depth of 3.5 centimeters of targeted tissues. All values of each participant were presented as difference (delta) from their pre-exercise baseline values. Since mental and physical disturbance can vary NIRS values, experimenters paid special attention to calm participants down completely until a stable baseline was established for 1 min before recording. The pre-exercise baseline is defined by minimal fluctuations in oxygenation (% oxygen saturation) and total hemoglobin without an obvious upward or downward trend for 1 min before cycling.

### 2.7. Cycling time to exhaustion

Endurance performance was assessed at 80% VO_2max_ following 5 min warm-up cycling (100 watts). A familiarization trial was conducted a week before the test. The criteria of exhaustion (cycling fatigue) failed to maintain 60 RPM for>10 s, heart rate>95% HR_max_, and rating of the perceived exertion (RPE) > 9. HR_max_ was estimated by subtracting age from 220. To obtain maximal aerobic capacity (cycling power at VO_2max_) of each participant, a graded exercise to exhaustion test protocol was performed on an electronically braked cycloergometer (894E, Monark, Varberg, Sweden) after a familiarization trial, 2 weeks before the performance test. VO_2_ and VCO_2_ were measured by a gas analyzer (MetaMax 3B Portable CPX System, CORTEX Biophysik, Leipzig, Germany). The initial workload started from 100 watt and incrementally elevated 25 watt every 3 min until volitional exhaustion. Criteria to obtain VO_2max_ required to achieve two of four conditions including: failed to maintain 60 RPM for>10 s, RER≥1.15, heart rate>95% HRmax, and RPE>9. We used modification of Borg CR-10 Scale RPE scaled from 0 to 10 to monitor the difficulty of the exercise at the end of each exercise challenge [11].

### 2.8. Statistical analysis

All data are expressed as mean ± standard error (SE). Performance data of each participant was expressed into delta (defecated minus non-defecated values). Significant difference between two conditions was compared using transformed data. For comparison between non-defecated and defecated conditions, all optical data of participants (10 values per second) were transformed into 20 averaged delta values (against pre-exercise baseline) during the entire cycling period. Pre-exercise baseline was the average of optical values (oxygenation and total hemoglobin) of 90 seconds. A pair *t*-test was used to distinguish the difference between interventions (non-defecated vs. defecated) and compared against pre-exercise baseline values. A minimal of four samples are sufficient to reach a power value of 0.9 for evaluating treatment difference in endurance performance. To reflect effect size of intervention, Cohen’s *d* was used, in which *d* = 0.2 considered a small effect size; *d* = 0.5 considered a medium effect size; *d* = 0.8 considered a large effect size; *d* > 1.2 considered a very large effect size. Type 1 error<5 % was considered significant and Type 1 error between 5–10% was considered moderately significant. All analyses were performed using SPSS statistics software (version 25; IBM Corporation, USA).

## 3. Results

**Figure 2.**
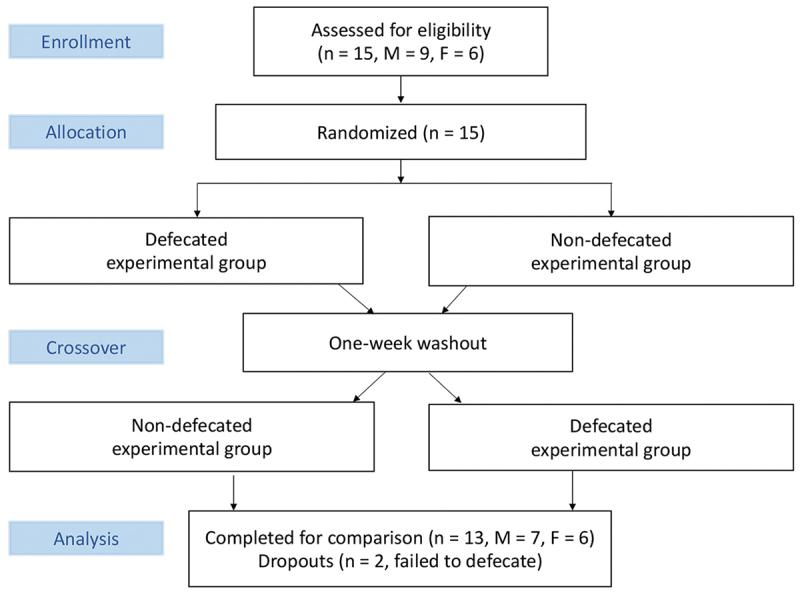
Flowchart of the study.

Figure 2 illustrates the flowchart of the study. Endurance performance (cycling time to exhaustion at 80% VO_2max_) was assessed 90 min after defecation and compared to non-defecated condition in a counter-balanced crossover manner separated by one week. Tissue oxygenation (% oxyhemoglobin to total hemoglobin) and blood distribution (total hemoglobin) in the cerebral and sub-navel regions were measured during the cycling performance test.

**Figure 3.**
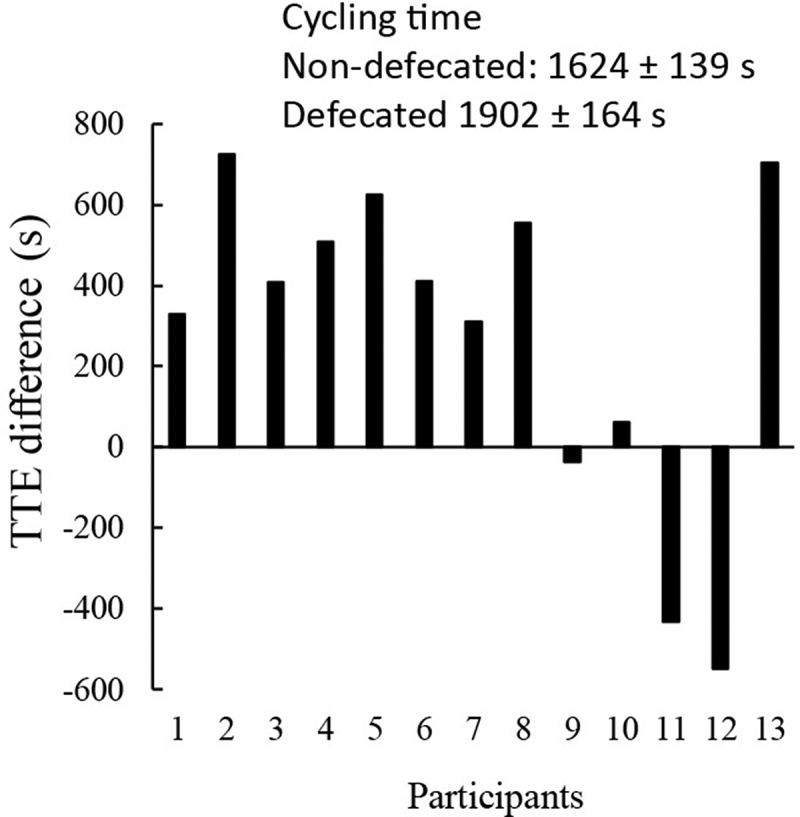
Defecation improved high-intensity cycling performance. Ten out of thirteen triathletes showed improved cycling time-to-exhaustion test (TTE) at 80% VO_2max_ following defecation (TTE difference: defecated minus non-defecated in second) by~17%. Endurance performance was conducted 90 min (0730 am) after the defecation at 18.5 Celsius degree and 60% humidity following a 10-h overnight fasting. Abbreviation: P, participant. Male: P2, P3, P4, P5, P8, P9, P11; Female: P1, P6, P7, P10, P12, P13. Values are presented as mean ± standard error.

Blood pressure and pulse wave velocity were measured to mirror autonomic nervous activity 10 min after defecation. Defecation slightly lowered systolic blood pressure from 115 to 111 mmHg (*p* < 0.05, *d* = 0.71) and diastolic pressure from 65 to 61 mmHg (*p* < 0.05, *d* = 0.71) compared with non-defecated condition. Decreased arterial stiffness (1047 ± 22 cm/s to 1009 ± 31 cm/s) after defecation was not statistically significant. Endurance performance (cycling time to exhaustion) was improved from 1624 ± 139 s to 1902 ± 164 s (*p* < 0.05, *d* = 0.51) after defecation (Figure 3). Nine out of thirteen participants demonstrated substantial improvements after defecation with two non-responders and two opposing cases.

**Figure 4.**
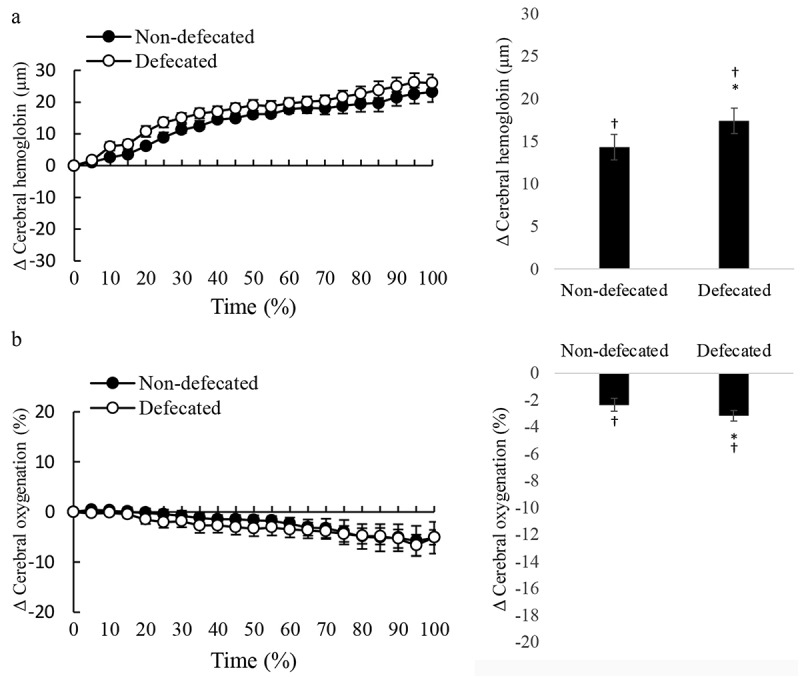
Defecation enhanced blood supply to prefrontal cerebral region during exercise. Cycling at 80% VO_2max_ decreased cerebral oxygenation (oxyhemoglobin to total hemoglobin ratio) from pre-exercise level for both defecated and non-defecated conditions (*p* < 0.01) (A: left panel, total hemoglobin trajectory line; right panel, average change of entire cycling period from baseline). Cerebral blood distribution in the prefrontal brain increased to compensate the deoxygenation during cycling (B: left panel, total oxygenation trajectory line; right panel, average change of entire cycling period from baseline). Since each triathlete had different cycling time to fatigue, time is displayed in a relative scale (% time completed). Values are presented as mean ± standard error. *Significant difference from Non-defecated condition, *p* < 0.05. ^†^Significant difference from pre-exercise baseline, *p* < 0.05.

During cycling, NIRS optical detectors were positioned in the centers of sub-navel region and prefrontal brain where the bright signals (high glucose utilization) of PET scan were located (described in the Methods and Figure 1). Real-time changes of cerebral oxygenation and blood distribution in the prefrontal brain during the cycling time to exhaustion trial are shown in Figure 4. Since all participants completed the cycling test for different durations, the results are presented in percent time to completion for both oxygenation and blood distribution. For comparison between non-defecated and defecated conditions, values of oxygenation and total hemoglobin of participants detected at 10 Hz were transformed to delta values (changes from pre-exercise baseline) and produced 20 averaged values (left panel). Average changes are shown by bar chart in the right panel. Cycling exercise decreased cerebral oxygenation progressively by~5% at exhaustion (Figure 4A) and increased cerebral blood distribution since the beginning of the exercise (Figure 4B). Cerebral deoxygenation was more pronounced during the last quarter period of cycling before exhaustion. While defecation delayed cycling time to fatigue, participants under defecated and non-defecated conditions reached similar levels of deoxygenation at the time when exercise can no longer be persisted. Defecation was found to increase more blood to the prefrontal brain region compared to levels without defecation during the first quarter and persisted throughout the cycling period.

**Figure 5.**
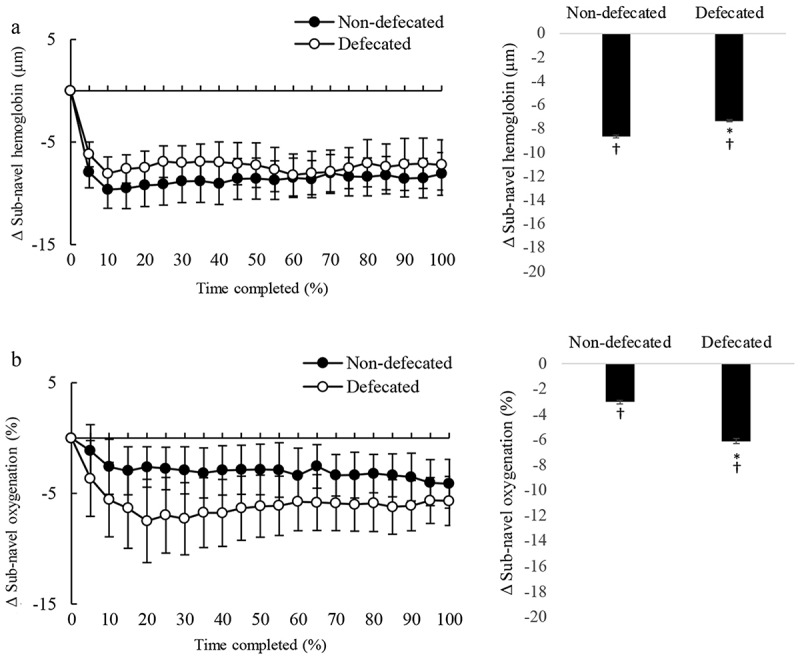
Defecation enhanced oxygen consumption in the sub-navel region. Blood distribution (total hemoglobin) in sub-navel region decreased from pre-exercise level (*p* < 0.05) to a similar extent for defecated and non-defecated conditions (A: left panel, total hemoglobin trajectory line; right panel, average change of entire exercise period from baseline). Defecation further lowered the decreased sub-navel oxygenation (oxyhemoglobin to total hemoglobin ratio) induced by cycling (*p* < 0.01) (A: left panel, oxygenation trajectory line; right panel, average change of entire cycling period from baseline). Since each triathlete had different cycling time to fatigue, time is displayed in a relative scale (% time completed). Values are presented as mean ± standard error. *Significant difference from Non-defecated condition, *p* < 0.05. ^†^Significant difference from pre-exercise baseline, *p* < 0.05.

During the cycling time to exhaustion trial, real-time changes of tissue oxygenation and blood distribution in the sub-navel region are shown in Figure 5. Defecation resulted in greater decreased sub-navel oxygenation during cycling than non-defecated conditions (Figure 5A), whereas the reduction in sub-navel blood distribution was comparable between defecated and non-defecated conditions (Figure 5B).

## 4. Discussion

Neuronal activation from the prefrontal cortex for motor unit recruitment is essential to maintain high-intensity exercise [1], which requires sufficient supply of blood to maintain cerebral oxygen. Rectal distension stimulates the autonomic nervous system in the brain [6] and thus we hypothesized a reduced competition for blood between prefrontal brain and enteric nervous system after defecation. The key findings of the study are: 1) Defecation resulted in greater blood pooling to the prefrontal brain during exercise than non-defecated condition; 2) Pre-exercise defecation significantly improved high-intensity endurance performance; 3) Defecation increased sub-navel oxygen consumption during exercise than non-defecated levels, evidenced by significantly lower oxygenation at similar levels of blood distribution; 4) Decreased blood pressure after defecation suggests a decreased regulatory burden to the autonomic nervous activity. The results of the study suggest that the improved endurance performance of elite triathletes after defecation is associated with blood-sparing effect to the prefrontal brain.

This study also supports the view that cerebral oxygenation is a rate-limiting determinant for sustaining high intensity exercise. It has been reported that the magnitude of cerebral oxygenation declines during cycling is intensity dependent [12], explaining why the high-intensity cycling is less durable compared with low-intensity cycling. In this study, the progressive decline in cerebral oxygenation during cycling at 80% VO_2max_ with a compensatory increase in cerebral blood distribution suggests an increased anaerobic metabolism in the prefrontal brain. The cycling exercise at this intensity was difficult to persist when cerebral oxygenation reached~5% below the pre-exercise level. Taken together, the improved endurance performance after defecation is associated with gaining capability to supply greater amount of blood to the prefrontal brain for compensating oxygen utilization, which explains the delayed falls of cerebral oxygenation concurrent with the extended exercise duration.

Exercise with fecal storage in the rectum may lead to competition for blood between the motor center in the central nervous system and autonomic nervous system within the brain. Rectal distension is known to increase blood pressure and heart rate [13] and increases oxygen demands in several brain regions for autonomic regulation apart from the prefrontal motor cortex [14]. A functional MRI study has shown that rectal distension activates greater brain regions in addition to the prefrontal cortex [15]. This will directly influence on the amount of blood distributed to the pre-frontal region. The gut has a high density of neural network containing the largest component of the autonomic nervous system connected to the central nervous system [16]. Therefore, the blood-sparing effect to prefrontal cortex from the brain regions responsible for regulating autonomic nervous activity is likely to explain the observed performance enhancement effect of defecation.

The study uncovered an unexpected finding, which showed that defecation increased sub-navel oxygen consumption during exercise evidenced by a substantial deoxygenation. This change was much smaller under non-defecated conditions. This observation implicates an unknown physiological role of sub-navel region for sustaining muscle contraction in humans. Oxygenation reflected by oxyhemoglobin to total hemoglobin ratio is mainly dictated by production rate of tissue carbon dioxide and hydrogen ion (the Bohr effect) in tissues [17] and autoregulation of blood vessel [18]. While the physiological significance of increased sub-navel region again remains elusive, the sub-navel region in the human body is highly metabolically active based on the PET scan image showing high glucose uptake. Interestingly, this site has been referred to as Dantian in ancient Chinese as the center where spirit is stored. Dantian was firstly described in Huang Di Nei Jing (Su Wen) [19] and the exact location of 2–3 inches below the navel was reported in a Chinese medical literature Baopuzi [20].

The major limitation of the study is its lack of comprehensive measurement to show the blood distribution in the entire human body during metabolic activity in different tissues and brain areas. Enhanced time to exhaustion could be a matter of GI distress that burdens autonomic nervous system. Despite this, the findings emphasize the crucial role of blood allocation to the prefrontal brain in maintaining high-intensity endurance performance. To further investigate this concept, tracing hemoglobin in multiple parts of the body, including the skin, heart, muscle, gut, and various brain regions, could provide a more accurate understanding of the effect of stool in the rectum on the central command from the prefrontal cortex during high-intensity endurance exercise and if this effect is influenced by other parts of the body.

## 5. Conclusion

This study demonstrated a performance enhancing effect of defecation for elite triathletes. This favorable outcome is associated with pooling more blood for the prefrontal brain to compensate increased oxygen utilization during high-intensity cycling. Decreased blood pressure after defecation suggests an alleviated burden for regulating autonomic nervous activity, allowing efficient blood allocation towards the prefrontal brain region to sustain muscle contractions. The role of increased sub-navel oxygen consumption during exercise after defecation requires further investigation.

## Data Availability

Data is available upon reasonable request.
